# Toxicity Effects of Functionalized Quantum Dots, Gold and Polystyrene Nanoparticles on Target Aquatic Biological Models: A Review

**DOI:** 10.3390/molecules22091439

**Published:** 2017-08-31

**Authors:** Giovanni Libralato, Emilia Galdiero, Annarita Falanga, Rosa Carotenuto, Elisabetta de Alteriis, Marco Guida

**Affiliations:** 1 Department of Biology, University of Naples Federico II, Complesso Universitario di Monte S. Angelo, via Cinthia ed. 7, 80126 Naples, Italy; giovanni.libralato@unina.it (G.L.); rosa.carotenuto@unina.it (R.C.); dealteri@unina.it (E.d.A.); marguida@unina.it (M.G.); 2 Department of Pharmacy, University of Naples Federico II, Via Mezzocannone 16, 80134 Naples, Italy; annarita.falanga@unina.it

**Keywords:** toxicity, nanosafety, nanoparticles, functionalization

## Abstract

Nano-based products are widespread in several sectors, including textiles, medical-products, cosmetics, paints and plastics. Nanosafety and safe-by-design are driving nanoparticle (NP) production and applications through NP functionalization (@NPs). Indeed, @NPs frequently present biological effects that differ from the parent material. This paper reviews the impact of quantum dots (QDs), gold nanoparticles (AuNPs), and polystyrene-cored NPs (PSNPs), evidencing the role of NP functionalization in toxicity definition. Key biological models were taken into consideration for NP evaluation: *Saccharomyces cerevisiae*, fresh- (F) and saltwater (S) microalgae (*Raphidocelis subcapitata* (F), *Scenedesmus obliquus* (F) and *Chlorella* spp. (F), and *Phaeodactylum tricornutum* (S)), *Daphnia magna*, and *Xenopus laevis*. QDs are quite widespread in technological devices, and they are known to induce genotoxicity and oxidative stress that can drastically change according to the coating employed. For example, AuNPs are frequently functionalized with antimicrobial peptides, which is shown to both increase their activity and decrease the relative environmental toxicity. P-NPs are frequently coated with NH_2_^−^ for cationic and COOH^−^ for anionic surfaces, but when positively charged toxicity effects can be observed. Careful assessment of functionalized and non-functionalized NPs is compulsory to also understand their potential direct and indirect effects when the coating is removed or degraded.

## 1. Introduction

The broad applications of nanoparticles (NPs) in industry and common-use products are progressively introducing them into our environment. Engineered nanomaterials are created and used in various industrial and commercial sectors and applied for their peculiar features, such as their size which ranges between 1 and 100 nm for at least one dimension, their high surface area to volume ratio compared to their bulk forms and their capacity for agglomeration or dispersion [1,2,3].

NPs have recently attracted an enormous attention because of their potential applications in chemical, catalytic, electronic, optical, mechanical, magnetic and medical fields [4,5,6] and most of them are considered non-toxic, bio-safe and have been extensively used as drug carrier cosmetics, fillings in medical materials, antimicrobial materials, and contaminated land and sediment remediation agents [7].

The specific characteristics of NPs can promote their transfer into environmental compartments, which renders fundamental the knowledge of the bioavailability, degradability, reactivity and toxicity of nanomaterials [8,9]. Moreover, as the amount and diversity of nanomaterial applications increase, new emerging potential categories of environmental contaminants are coming out and evaluations of their release into the environment and their impact on natural ecosystems are necessary. Toxicity tests are key tools to evaluate hazard and risk assessments in order to identify potentially dangerous effects on human health and the environment. Unfortunately, toxicity studies are not always systematic due to the different chemical composition of the material tested, and the multiplicity of experimental approaches, sometimes leading to contradictory results [10]. Possible mechanisms mediating toxicity are not even investigated, and only results on toxicity assessment are reported. 

The toxicity of metallic NPs has been studied intensively during the last few years, as shown by the large number of publications on this subject [11]. Several studies are focused on the influence of the physico-chemical properties of metallic NPs (i.e., size, shape and surface coating) and on preparation methods that could change their toxicity effects on living organisms [12], yet conflicting and inconsistent results on the factors determining toxicity are commonly reported. 

NPs showed higher and unique toxicity compared to their corresponding bulk materials. Thus, it is very important to gain a better understanding of their mode of interaction, uptake, accumulation through generation and impact on the biosystems, and thus knowledge would help in defining control measures to avoid nano-pollution in ecological systems [13,14].

While overall knowledge on the potential toxicity of NPs is still lacking, researchers analyze different aspects, such as interaction with the immune system, perturbation of cell membranes, and transport of toxic portions that are emerging as potentially hazardous aspects of NPs. It is thus important to safely design NPs, in order to guarantee their safety throughout their life cycle while keeping the desired properties [11].

One key area of research is thus devoted to the improvement of stability, safety, and efficacy of NPs through binding to peptides or peptidomimetics. Modifications of the surface of NPs with peptides will allow a reduction of their toxicity, and an enhancement of stability, while perhaps also determining an improvement of the properties of the peptides. NPs can serve as innovative drug delivery systems for antimicrobial peptides (AMPs) [11,15] offering the possibility to target the delivery of AMPs to a specific site with controlled release overtime, thus minimizing side-effects and increasing efficacy also due to NPs potential multi-valency. Specifically, inorganic nanomaterials have attracted significant attention since they display their own antimicrobial activity, which may provide additive or synergistic effects when combined with AMPs [16].

Most of the current literature on the toxicity of NPs comes from studies on mammalian cells but it is very important to know their potential harmful effects on the environment as well as their genotoxic and potentially carcinogenic effects which should be identified to allow for their safe use. To date, NPs are believed to be possibly cytotoxic, genotoxic and carcinogenic to humans, and can cause other undesirable side effects [17], but concerns with their harmful effects on the environment and human health have been raised only recently. The frequent detection of NPs in the aquatic environment reflects a rapidly growing number of engineered nanoparticles being used and their incomplete removal during passage through sewage treatment plants and relatively high persistence in water matrices [18,19]. Particularly, hot spots of NPs could be present in hospital wastewater due to their ever more frequent use in medical applications for drug delivery (unpublished data).

Recent studies have confirmed that NPs are released into the environment, and in particular the aquatic environment. For example, significant concentrations of nano-Ag can be released from AgNP-containing textiles during washing [20], while contamination of sewage sludge with Ag and AgNPs has been detected [21], and 0.1 μg L^−1^ AgNPs has been detected in wastewater effluents [22]. ZnO NPs are among the most used NPs and consequently dispersed in the environment [23]. However, the potential ecotoxicological effects of these NPs on different organisms within the aquatic environment are still unclear. Although an increasing number of studies have demonstrated the bioavailability and toxicity of aqueous AgNPs in a variety of algae, invertebrate and fish species [24,25,26], such ecotoxicological research has mostly focused on the exposure of individual organisms. Since NPs could potentially accumulate within ecosystems and ultimately damage ‘top-level’ species, trophic accumulation and dietary transfer of NPs through the food chain are important issues that need to be studied too. An important question is the contribution to toxicity of dissolved ions; in fact, the total toxicity results from the toxicity of the NPs themselves (Tox_part_) and that of solubilized ions (Tox_ion_).

Xiao et al. [27] measured the acute toxicity of ZnO NPs in *Daphnia magna* and concluded that it was only 31% was due to Tox_ion_ and that the Tox_part_ played a dominant role, even if the total internal concentration of ZnO was proportional to the concentration of ZnO NPs administrated to daphnids. They concluded that this fact was due to the internalization of NPs and consequent dissolution of ions. Similarly, Bacchetta et al. [28,29] concluded that after a chronic exposure of daphnids to ZnO NPs and ZnSO_4_ toxicity is due both to accumulation and persistence of NPs into tissues and to ion dissolution.

Exposure of organisms to sublethal concentrations of toxic NPs has been observed to terminate in changes at cellular and biochemical levels. When NPs enter into an organism, their interaction with target sites or receptors can determine an increase in oxidative stress that can produce a proteomic or non-proteomic response [11]. These responses could involve the antioxidant defense system to protect the organisms against reactive oxygen species (ROS) (e.g., catalase (CAT), superoxide dismutase (SOD), glutathione peroxidase (GPX) or general detoxification enzymes like glutathione-S-transferse (GSTs) or stress proteins like metallothioneins (MT)). The presence of NPs can disturb steady-state conditions, leading to an increase of ROS production, a lack of defense capacity resulting in inactivation of some enzymes, oxidation of proteins, damage of DNA or some other molecules such as lipids. The use of oxidative stress biomarkers has become a promising tool to evaluate the toxic effects of NPs; in fact, they may disrupt the balance of biological oxidant-to-antioxidant ratio in aquatic species, leading to elevated levels of ROS and resulting in oxidative stress that can be revealed by transcription genes related to antioxidant system [30].

This review provides an overview of most recent studies about the impact of quantum dots (QDs), gold (AuNPs), and polystyrene-cored (PSNPs) NPs, evidencing the role of NP functionalization in toxicity definition. Particularly, our attention focused on some key biological models like *Saccharomyces cerevisiae*, fresh- (F) and saltwater (S) microalgae (*Raphidocelis subcapitata* (F), *Scenedesmus obliquus* (F) and *Chlorella* spp. (F), and *Phaeodactylum tricornutum* (S)), *Daphnia magna*, and *Xenopus laevis* as target biological models constituting a potential full battery of toxicity tests.

## 2. Quantum Dots, Gold, and Polystyrene NPs

### 2.1. Quantum Dots Nanoparticles (QDs NP)

Manufactured nanomaterials (MNs) have attracted great attention in several areas of science and engineering, for their potential applications in many industrial areas. Among MNPs, quantum dots (QDs) are some of the most used and characterized. QDs are semiconductor crystals of nanometer dimensions (2–10 nm), containing 200–10,000 atoms, which form an important class of fluorescent labels for biological, biomedical and bio-sensing applications [31]. Due to their very small size and their unique photochemical and photophysical properties, QDs have shown excellent fluorescence properties, including high brightness, photostability, tunable emission spectra and a high, specific bioactivity when coated and linked to functional groups. Nevertheless, little is known about the health risks from exposure to these NPs and above all, their large scale diffusion in the environment has led to alarming speculations concerning their potential long-term toxicity related to the fact that these materials contain heavy metals such as Cd, As, Zn, Pb. The main consequences of exposure to MNPs are the occurrence of ROS genotoxicity and apoptosis due to mitochondrial damage and metallic ion production [32]. CdSe QDs are often used for biological studies because they are easily prepared, have size-tunable properties, a narrow emission band and a large absorption spectrum. They can be coated with ZnS in order to protect the core from oxidation and other degradation processes that could release Cd ions into the medium. Their composition and size confer them several advantages in imaging. Resistance to photobleaching provides better confocal images, while the relative stability allows for, in contrast to other MNPs, particle uptake to be separated from uptake of dissolved particle components. Their electron-dense nature makes them easy to see using electron microscopy and the particle size and shape can aid in their identification within tissues. The high fluorescence of these NPs allows extremely low loading concentrations, so acute toxicity is avoided. Thanks to their high volume ratio, they can be conjugated with multiple ligands and are useful as imaging probes in various tumors [33].

QDs’ toxicity depends on multiple factors due to the individual QD physiochemical properties and environmental conditions. QD toxicity was shown to be influenced by their size, charge, concentration, outer coating bioactivity (capping material, functional groups) and oxidative, photolytic and mechanical stability. Two major mechanisms are involved in the toxicity effects: Cd^2+^ in the structure that could cause interference in DNA repair or increase of oxidative stress and free radical formation. It is known that the release of Cd^2+^ from the core of QDs influences their toxicity, but also that QDs can aggregate to form membrane structures, inducing ROS generation that were demonstrated to cause DNA nicking and breaks, metabolic function loss and cell apoptosis [34,35]. Many researchers are expressing doubts about the safety of using QDs for treating patients, as there are not enough reliable studies on their toxicological effects.

### 2.2. Gold Nanoparticles (AuNPs)

AuNPs have received great attention because of their unique characteristics that make them suitable for a series of application in the biomedical field such as diagnostics and therapeutics [36], drug delivery and cancer treatment [37]. This increased use has led to a diffusion of AuNPs into the environment, which represents a hazard for aquatic ecosystems. Cytotoxic effects of AuNPs were identified in several mammalian cell lines, and different toxic effects have been reported and correlated to size, shape and coating but also to cell lines or murine models used [38,39]. The results are conflicting and some reports suggest that in vivo and in vitro effects are not comparable. In fact, some authors state that AuNPs are toxic to certain organisms, while others that they are not toxic to the same organisms [40,41]. Thus, it is important to investigate the ecotoxicity of AuNPs both in their functionalized and non-functionalized forms using toxicity tests to provide a background for ecotoxicological hazard assessment. Functionalized AuNPs have attracted attention for imaging, diagnosis and delivery of antibacterial drugs because NPs not only protect the drug from the external environment, but will also concentrate it at the desired infection site with doses higher than those reachable with the administration of free molecules. AuNPs can be easily functionalized with a great variety of AMPs with an improvement of their activity and a decrease of environmental toxicity.

### 2.3. Polystyrene Nanoparticles (PSNPs)

Recently, PSNPs, incorporating fluorescent dyes and polymer matrixes with size ranging from 50 to 300 nm, have attracted much interest because of their special properties, namely their nanoscale size, uniform shape, and narrow size distribution. Therefore, they find a wide range of applications in various fields such as in optical sensor, biomedicine, food, textiles and paint coating.

Polystyrene is known to be one of the most heavily used polymers in our routine daily activities. Amongst others, it is commonly used in various food industries as food containers, storage, and carriers as the USDA has approved its use. PSNPs could be potentially strong pollutants of aquatic environments that act as a sink for NPs. The potential toxic effects of PSNPs on aquatic organisms deserve full attention [42]. PSNPs may in fact constitute a serious danger, not only for these organisms, but also for their consequent domino effects, in particular along the aquatic food chain [43]. These NPs can be divided into three main groups according to their effective surface charge: cationic, anionic or neutral (unmodified) PSNPs. Their surface charge will depend on the surface coating, the most common functional groups used being NH_2_^−^ for cationic, and COOH^−^ for anionic surfaces. This allows them to pass more easily through the cell membrane, as they share a similar molecular structure to proteins, rendering them a potential tool for drug delivery. However, some studies have shown that, while the neutral and negatively charged PSNPs are considered to be not toxic, the positively charged PSNPs induce some toxicity [44]. Positively charged NPs adsorb more on cell membranes and show higher level of internalizations when compared with negatively charged or neutral NPs. NPs encounter a large variety of biomolecules in vivo, where non-specific adsorptions can potentially alter the physicochemical properties of the NPs [45]. Studies with model polystyrene latex NPs of varying nanosizes (50 and 100 nm) and surface chemistry—unmodified, amine-modified and carboxyl-modified—have shown that they are able to induce platelet aggregation in vitro, suggesting a poorly defined potential mechanism of increased cardiovascular risk upon NP exposure [46,47]. Hardy et al. [48] investigated the immunological imprints of uncoated polystyrene NPs of 50 nm and 500 nm, evidencing that these particles imprint a differentially modulated induction of acute allergic airways inflammation, with 50 nm significantly inhibiting adaptive allergen-specific immunity. Recent literature on airway cells [49], intestinal epithelium [50] and blood coagulation [51] concluded that their activity and different ways was dependent on the PSNPs’ size and surface chemistry and this should be taken in account before using PSNPs for medical treatments. Tiago dos Santos et al. [52] investigated NP uptake mechanisms in a range of representative human cell lines, including HeLa (cervical cancer), A549 (lung carcinoma) and 1321N1 (brain astrocytoma). They performed uptake experiments using carboxylate PSNPs of 40 nm and 200 nm diameters, as model NPs of sizes comparable to typical endocytic cargoes. Their results clearly indicated that, in all cases and cell types, PSNPs entered cells via active energy-dependent processes. They suggested that the same NPs might exploit different uptake mechanisms to enter different cell types. Oral exposure to polystyrene NPs affects iron absorption [50]. PSNPs of 44 nm accumulate rapidly and more efficiently in the cytoplasm of adenocarcinoma cells compared to PSNPs of 100 nm; both PSNPs showed an energy dependent mechanism of internalization and a clathrin-mediated endocytosis pathway. Dose response treatments revealed a non-linear curve. PSNPs also affected cell viability, inflammatory gene expression and cell morphology. PSNPs of 44 nm strongly induced an up-regulation of IL-6 and IL-8 genes, two of the most important cytokines involved in gastric pathologies [53]. Studies carried out on embryos showed that fluorescent PSNPs up to 240 nm were able to cross the placental barrier [54] 17 and 20 nm carboxylic PS distribute in the embryonic and extra-embryonic germ layers of ectoderm, mesoderm, and endoderm. When the particles are bigger than 100 nm, they accumulate in extra-embryonic tissue. A growth inhibition was observed in the embryos containing NPs [55]. In contrast the studies of Bosman et al. [56] showed that exposure to polystyrene-based NPs at the concentration tested are not associated with embryonic loss. However, it should be mentioned that the observations made on contact embryos where the PSNPs might have been modified through the transport process from the extracellular to the intracellular environment may represent more valid information for environmental studies than observations made through directly injected PSNPs [57]. Guarnieri et al. [58] showed that the fate of the gH625- functionalized PSNPs depend on the mechanism of translocation of gH625 a membranotrope peptide that have a relevant impact on the nanotoxicological profile of positively charged NPs reducing NPs toxicity. Finally PSNPs are used as carriers for antimicrobial peptides. Polystyrene NPs modified with vancomycin-antibody were produced to highlight the power of utilizing small molecule probes for the capture of biomolecules [59]. Moreover polystyrene sulphonate assembled with polymyxin B was utilized for the delivery of this antimicrobial peptide currently used in the clinic as a last resort antibiotic against multidrug-resistant Gram-negative bacteria [60].

## 3. Toxicity on Target Biological Models

### 3.1. Saccharomyces cerevisiae

The unicellular eukaryote *Saccharomyces cerevisiae*, a well-established model organism in basic research and a workhorse in traditional and innovative biotechnological processes, has been used in ecotoxicology as an alternative to other more commonly used bioindicators, due to its lack of pathogenicity, relative simplicity, possibility to be easily cultured in controlled conditions, availability of experimental tools, protocols, database, and genome-wide-methodologies [61].

Nevertheless, so far relatively few reports on the eco-toxicological impact of nanomaterials on *S. cerevisiae* have been published. Various metal oxide (CuO, TiO_2_, ZnO_2_, FeO) NPs and fullerene have shown a very low toxicity on yeast [62,63,64,65,66].

The standard toxicity test consists in the determination of yeast growth inhibition, by incubating the cells in culture medium in the presence of different NP concentrations and measuring growth rates and/or biomass yields. In some cases, the median inhibitory concentration (IC_50_) is reported. In addition, cell viability or survival is often evaluated.

In 2012, a first investigation on QDs toxicity towards *S. cerevisiae* [67] was published. Water soluble *N*-acetylcysteine (NAC)-capped cadmium telluride (CdTe) QDs of different size with green or orange emission were synthetized and their toxicity against yeast was tested with a combination of microcalorimetric, spectroscopic, electrochemical and microscopic methods. A microcalorimetic technique was used to record the heat flow rate of yeast growth reaction, allowing to calculate the growth rate constant of yeast exposed to different CdTe QDs concentrations versus that of the control and consequently determining IC_50_. Determination of cell viability directly demonstrated the toxicity of QDs by microscopic counting. Confocal laser scanning microscopy and transmission electron microscopy demonstrated that CdTe QDs ranging from 4.1 to 5.8 nm were internalized and localized in the nucleus., so cytotoxicity mechanisms were ascribed not only to Cd ions released by the NP, but also by the direct interactions with cellular components, including the cell surface.

The same research group [68], with an analogous approach, compared the biological effects of CdSe QDs and CdSe QDs coated with ZnS, both of which orange emitting with a similar size of about 3 nm. The micro-calorimetric analysis of growth showed that, once coated with a ZnS shell, CdSe QDs had a negligible toxicity. Again, the role of released Cd ions was ascertained in determining toxicity, but microscopic studies revealed that CdSe QDs adhered to the surface of yeast cells and also reduce mitochondrial membrane potential.

Also, in their investigation on the effects of large CdSe QDs (20–30 nm) on *S. cerevisiae*, Sun et al. [69] did not solely attribute the toxicity mechanisms to released Cd ions, but also found accumulation of ROS and enhancement of vacuolar membrane permeabilization, whereas a reduction in mitochondrial membrane potential was not detected.

Since it is reported that activation of autophagy is common in mammalian cells when exposed to QDs, a deeper investigation on CdTe QDs toxicity on *S. cerevisiae* [70] aimed to explore the possible role of autophagy in mediating the already ascertained toxicity of these nanomaterials on yeast [67]. Transmission electron microscopy and green fluorescent protein tagged Atg8 processing assay [71] revealed that inhibition of autophagy at late stages contributed to orange emitting CdTeQDs’ toxicity in yeast. This contrasting result with respect to mammalian cells was explained by the authors with the observation that in mammalian cells accumulation of autophagosome may derive from blockage of autophagic flux rather than induction of autophagy [72].

In 2016, the first toxicogenomic analysis on the effects of QDs on *S. cerevisiae* was published [73]. Screening of a genome-wide deletion mutant collection [74] was exploited to provide the genetic basis for resistance to CdS QDs exposure. Several metabolic processes, including abiotic stress response, mitochondrial organization, transport, and DNA repair resulted related to the cellular response to CdS QDs.

In [75], the same research group carried out a more detailed investigation on the yeast response to CdS QDs (in conditions of limited lethality (<10%)), by a transcriptomic analysis accompanied by a series of physiological and microscopic characterizations. They identified mitochondrion organization as the leading functional category for the genes affected by CdS QDs. They also observed a reduction in oxygen consumption, cytochrome content, and mitochondrial membrane potential. Interestingly, the authors concluded that *S. cerevisiae*, being a facultative anaerobe, is the ideal organism for studying the effects of toxicants in conditions which shut-off mitochondrial function. This allows correlating genes with human orthologs in a comparison, which allows one to predict the mode of action of similar toxic compounds in humans.

As regards the chronic exposure of *S. cerevisiae* to nanomaterials, a recent finding [76] demonstrates that a *S. cerevisiae* population evolved to resist to CdSe QD toxicity. In fact, yeast grew normally after constant exposure for 24 days to CdSe QDs at half minimum inhibitory concentration of the progenitor. Whole-genome sequencing and single nucleotide polymorphism (SNP) analysis evidenced that the ubiquitin ligase gene, probably involved in Cd ions tolerance, was mutated in the evolved population.

Reports on the effects of gold and polystyrene NPs and their derivatives, on *S. cerevisiae* are relatively scarce. Faezali et al. [77] investigated the toxicity towards *S. cerevisiae* of a newly synthesized gold complex, namely trichloro(2,4,6-trimethylpyridine)Au(III), intended to be used as an anticancer agent. The complex was grafted on nanoporous silica, MCM-41, to enhance the efficiency of uptake by mammalian cells and prevent aggregation. The authors demonstrated that the grafting of trichloro(2,4,6-trimethylpyridine)Au(III) on MCM-41 dropped the IC_50_ about 50-fold to an IC_50_ of 0.25 mM.

A more detailed analysis is due to Smith et al. [78] that investigated if the use of yeast model could be informative of the possible toxicity of relatively small (0.8–1.0) atom gold core NPs functionalized with positively charged *N*,*N*,*N* trimethylammoniumethanethiol (TMAT), negatively charged 2-mercaptoethanesulfonate (MES) or (iii) neutral 2-[2-(2-mercaptoethoxy)ethoxy]ethanol (MEEE). No effects on growth were found in treated versus control cells, and positively charged NPs reduced the yeast survival of stationary cells. The authors did not attribute the survival reduction to a mechanism involving endocytosis, but, in the absence of uptake, to a distortion of a membrane potassium channel, presumably leading to leakage of cytoplasmic material. The observation that a *trkD* mutant (*TRK1* encodes a potassium channel component) was resistant to NP exposure supported this hypothesis. Indeed, for the first time in the assessment of nanotoxicity toward *S. cerevisiae*, Smith et al. [78] used the screening of a non-essential yeast deletion library [74] to determine which gene might predispose yeast to damage. Resistant mutants were recognized and a GO (gene ontology) analysis allowed identifying not only the *trk* mutant, but also 16 respiration-deficient mutants. Therefore, also functionality of mitochondria was suggested to mediate toxicity.

Larger AuNPs (5 nm-diameter) alone or functionalized with the antimicrobial peptide indolicidin, a novel complex proposed by [79] to stabilize indolicidin in the perspective of its biomedical applications, affect neither growth nor viability of *S. cerevisiae* cells. Instead, they exerted a genotoxic effect, as evaluated by alkaline comet assay [80], accompanied by indicators of oxidative stress response. A progressive reduction of this genotoxic effect was observed after 72 h exposure, presumably due to the activation of DNA repair mechanisms.

This finding was in agreement with the general observation that *S. cerevisiae* growth results scarcely affected by exposure to gold nanomaterials, whereas genotoxic effects and consequent DNA repair have been recognized by using both the conventional phenotypic alkaline comet test [81] and a newly developed quantitative toxicogenomics assay, that detects and quantifies molecular level changes in the regulation of DNA damage repair pathways.

Nomura et al. [82] have explored the toxicity towards *S. cerevisiae* of different polystyrene latex NPs (PLSNP) labeled with fluorophores non-modified and modified with various functional groups (amine, carboxyl, sulfate). Toxicity tests consisted in the assessment of cell viability by colony count following exposure to the different NPs dispersed in NaCl, and the behavior of PLSNP was studied by confocal laser scanning microscopy. The authors found that only positively charged amine-modified PLSNP were toxic, and related toxicity to the ionic strength of the solution. At high ionic strength, amine PLSNP were internalized by endocytosis and resulted not toxic, whereas at low ionic strength they adhered on the surface of dead cells. Apparently, the increased electrostatic force between PLSNP and cell surface exceeded the uptake rate of NPs into the cells, resulting in their accumulation on the cell surface, this possibly decreasing membrane fluidity. 

### 3.2. Microalgae

Morelli et al. [83] studied the potential toxicity of QDs on the marine microalga *P. tricornutum* because the stability of NPs in sea water is an important requisite for efficient interactions with living organisms. They found no toxicity at low concentrations while at high concentrations a dose dependent inhibition of growth rate and a concomitant increase production of ROS and SOD and CAT activities were found.

Worms et al. [84] studied the effect of carboxyl-functionalized polymer coated CdSe/ZnS QDs on three different green algae with different cell wall characteristics (*Chlorella kesslerii*, *C. reinhardii* and a cell wall-less strain of *C. reinhardii*) concluding that carboxyl-QDs could influence metal bioavailability due to the interactions of NPs with cell wall.

Oukarroum et al. [85] showed that toxicity and uptake of nitrogen oxide NPs (NO-NPs) on *C. vulgaris* increased for the formation of agglomerates that adsorbed at the surface of algal cells entrapping them and causing a reduction of availability of light and nutrients necessary for photosynthesis and cellular division. Similar results were also found when algal cells of *R. subcapitata*, *Chlorella* sp. and *Scenedesmus* sp. were exposed to SiO_2_ and TiO_2_ NPs.

Toxicity increased with a direct correlation to the increasing exposure concentration of NPs suspension. The release of Ni^2+^ from NiO-NPs represents one possible mechanism of toxicity because TEM analysis confirmed that Ni^2+^ were released from NiO-NPs inside the cell after bioaccumulation.

Nolte et al. [86] investigated the influence of polystyrene and AuNPs surface functionalization on green alga *P. subcapitata* utilizing a combination of method that allow a detailed evaluation of the algal toxicity by NPs. They showed an increased adsorption of functionalized PSNPs to the algal cells explained by change in cell wall and zeta potential.

Hoecke et al. [87] reported the ecotoxic effect of two types of polymer-coated AuNPs on *P. subcapitata*. In aquatic environments, agglomeration/aggregation of AuNPs is a common fate that does not indicate a decrease of toxicity but sometimes the aggregation could facilitate ingestion. Gilroy et al. [88] established potential transfer of AuNPs in the food webs and they stated that most NPs remained in the digestive tract did not affect reproduction and was eliminated with ejection.

Iswarya et al. [89] explored the impact of Zn^2+^ present in the freshwater environment at an average concentration of <0.05 mg/L, on the toxicity of AuNPs with different size and different surface-capped (citrate and PVP) on the green alga *Scenedesmus obliquus*. They found that as the concentration of AuNPs increased, the relative toxicity of algal cells was also found to increase for all the types of AuNPs tested with respect to the control. Differences were found only in different capping agent on the surface with more toxicity in citrate-capped AuNPs, and in this case also size depending. It is also confirmed that zinc ions showed an antagonistic ability to interfere with the toxicity caused by AuNPs on green algae *Scenedemus sp.* independently from the surface capping, then is difficult to attribute the effective toxicity to them.

Galdiero et al. [90] studied the biological effects of melittin, an antimicrobial peptide well-known, on *R. subcapitata* showing an inhibition of growth for the algae also at low concentration. As concerns genotoxicity of melittin *R. subcapitata* showed DNA damage in comparison to untreated control samples.

### 3.3. Daphnia magna 

Studies on the toxic effects of NPs on aquatic organisms are mainly focused on metal and metal oxides such as silver, copper, titanium and zinc oxides [91,92,93]; nonetheless it is very important to know which are the ligands on their surface because they could change the surface properties of NPs and as a consequence their interactions with the environment. Another important feature is the aggregation, which is key for toxicity because the change of charge of the surface of NPs can promote or prevent aggregation and consequently the uptake by organisms.

To date the effect of QDs on the biological environment is not fully understood. Most studies assess the toxicity of QDs to secondary consumers such as *D. magna*, a freshwater invertebrate. Studies showed that negatively charged QDs are taken up by daphnids better than neutral or positively charged QDs [94]. In vitro experiments have demonstrated that cell toxicity is due to intracellular QDs localization with consequent release of Cd^2+^ into cytoplasm and ROS generation, instead in vivo toxicity has been evaluated by viability and sublethal toxicity in organisms such as algae, crustaceans, amphibians, and zebra fish.

Being the toxicity the major limitation for applications of QDs, in order to solve this problem, QDs have been functionalized by molecules like antibodies, peptides or proteins [95].

Lee et al. [96] investigated how exposure to different surface coated CdSe/ZnS QDs influence *D. magna*. Unmodified QDs are eco-toxic by forming reactive oxygen species and DNA damage in *D. magna*, so a proper surface coating is key to avoid their adverse side effects. However, QDs with various coatings could undergo modifications in the environment and could then become toxic. They reported that three different QDs coated with polyethylene (QEI), amphilic polymer with amine (QSA), amphilic polymer with carboxylic (QSA) showed different properties and in the environmental system these properties are subjected to transformation by external factors, such as aquatic chemistry, which results in different toxicological dynamic conditions.

Kim et al. [21] showed that QDs with surface coating of 3-mercaptopropionic acid (MPA) and tris-*n-*octylphophine oxide/gum arabic) under environmentally relevant levels of UV-B light caused an increase in mortality and an increase ROS generation in a dose dependent manner in *D. magna*. Several studies reported that UV could increase cytotoxicity of QDs [23] and under photolytic conditions QD core metals could be released being toxic to *D. magna* also at low concentrations.

Chen et al. [97] functionalized the antimicrobial peptide UBI 29-41, originally isolated from mouse macrophage cells with specificity for targeted bacterial cells, with ZnO QDs (ZnO@BSA-PEP-MPA) showing an improved antimicrobial activity with an increase of permeation of bacterial membranes with low bio-toxicity; results also supported that the complex could be utilized for anti-multidrug resistance applications. It was found that the main antimicrobial mechanism of NPs is to disrupt the bacterial cell membrane, which leads to the leakage of intracellular material and alteration of the membrane protein or enzyme activity in a ROS mediated manner.

Carillo-Carrion et al. [98] used a colistin-functionalized CdSe/ZnSQDs (Colis-QDs) to detect Gram-negative bacteria using a fluorescent marker, such as a bacteria sensor. Galdiero et al. [99] showed that the activity of QDs functionalized with the antimicrobial peptide indolicidin was improved compared to indolicidin alone and had a lower ecotoxicity when used in different tests on *D. magna*. In particular, the ecotoxicity of the complex QDs-indolicidin decreased showing a dose-response curve of immobilization; the ROS production was immediately balanced with enzyme activation with an increase of SOD and CAT even if there was DNA damage in both conditions. When the complex was used to test how multigenerational stressors influenced *D. magna* [100], it was shown that over three generations QDs-ind produced adverse effects on reproduction and developmental but also a genotoxic effect that was transmitted to the following generations and the expression of four genes Dhb, Vtg, CYP4, CYP314 was up-regulated in response to the hazardous effects of toxic compounds. Maselli et al. [100] showed multigenerational effects and DNA alterations on *D. magna* subjected to sublethal concentrations of the complex QDs-indolicidin. They found that the total amount of eggs, total number of brood and body lengths decreased along generations, DNA damage occurred during generations and produced expression alterations of Dhb, Vtg Cyp4 genes.

Galdiero et al. [101] investigated the uptake of functionalized QDs with the membranotropic peptide gH625 by *D. magna* to prove a specific intracellular localization with a low toxicity before starting experiments on mammals. In fact, it was shown that the complex gH625-QDs reached body compartments of the organism and was excreted in about 24 h, differently from QDs alone that persisted in organisms with a high toxicity. Also the ROS production with activation of enzymatic response and DNA damage were lower in the complex than in QDs alone.

Dominguez et al. [102] evaluated the effects of surface functionalization of AuNPs with different ligands on *D. magna*. They found that positively charged AuNPs-CTAB (cetyltrimethylammonium bromide) and AuNPs-PAH (polyallylamine hydrochloride) are more toxic than the negatively charged AuNPs-MPA (mercaptopropionic acid) and AuNPs-Cit (citrate).

Nasser et al. [103] studied the impact of polystyrene NPs functionalized with carboxylic acid (COOH) and amino (NH_2_) groups on the uptake, retention and toxicity of *D. magna*. They found that coated PS-NPs compared to uncoated agglomerate as a function of incubation time, increasing their size due to proteins released by *D. magna*. In fact, it is very important in order to understand the NPs toxicity on *D. magna* to dissect the role of secreted proteins that mediate toxicity. It is not surprising that the NH_2_-PS-NPs are more toxic than COOH-PS-NPs, both in presence or in absence of secreted protein, indicating that it is necessary to modify the Organization for Economic Cooperation and Development (OECD) tests to enhance the concept that removal and uptake into the organism need to be considered as an important part of bioaccumulation and consequently of toxicity.

### 3.4. Xenopus laevis

Exposing living organisms to NPs is potentially hazardous especially during embryogenesis thus this endpoint deserves full attention [104]. *X.* *laevis* is a consolidated biological model used for embryotoxicity studies associated to bioimaging [105].

Uncoated PSNPs of 48 nm administered during early stages of larval development of *X. laevis*, using either contact exposure or microinjections, are lethal depending on concentration, and affect embryonic development showing toxic/teratogenic effects. These effects may be due either to the amount of NPs that penetrate into the cells and/or the “corona” effect caused by the interaction of PSNPs with cytoplasm components [105]. Symens et al. [106] using the *Xenopus* nuclear envelope re-assembly assay, found that the nuclear enclosure of NPs were dependent on size and charge of the polystyrene beads. Stylianou and Skourides [107] assessed relatively long-term effects of NIR pulses on embryonic development through the tadpole stage. They developed parameters for near infrared (NIR) pulses that did not affect embryonic viability or morphology and delivered QDs as effectively as manual injection. Higher intensities of NIR pulses caused permanent damage to the targeted cells, and thus NIR pulses may also prove useful for ablation of specific cells within tissues. They are the first to report the use of NIR QD’s to image mesoderm migration in vivo with single cell resolution and provide quantitative in vivo data regarding migration rates. 

Mogi et al. [108], injected quantum dot nanocrystals and polystyrene beads in *X. laevis* embryos to study the development of larval brain ventricles and the patterns of cerebrospinal flow via an imaging assay. *X. laevis* embryos raised in Frog Embryo Teratogenesis Assay (FETAX)-*Xenopus* containing QDs or gH625-QDs showed that NPs localized in gills, lung and intestine with different distributions, indicating that the uptake of gH625-QDs was enhanced; the functionalized QDs had a significantly lower toxic effect on embryos’ survival and phenotypes [101].

## 4. Conclusions

This review about the toxicity of functionalized NPs on some key bioindicators evidences that surface properties can play a significant role in the toxicity effects of NPs conditioning exposure outcomes, also due to possible surface transformations mainly due to the aquatic chemistry.

Considering QDs, AuNPs, and PSNPs, it is really difficult to identify the best biological model and endpoint, because the sensitivity of species and the way NPs and their relative coatings can act are strongly dependent on the exposure conditions. Thus, results are highly species-specific and NPs-specific with no possibility to extract generalized conclusions. In general, PSNPs showed lower toxicity effects compared to AuNPs and especially to QDs. Indeed, QDs can contain Cd, Se and Te that can present very high toxicity levels also in their ionic form that can be generated once in contact with an aqueous medium. Existing knowledge gaps are mainly related to assessment of the toxicity of the functionalized coating and NPs are the safe-by-design approach concept is still missing. Moreover, targeted studies on toxicity tests carried out onto a broader range of marine, freshwater and terrestrial species. Such studies should also concern the effects caused by mixtures of different NPs. The focus on the effects of both a long-term exposure (chronic stress) and a low level exposure could be relevant to obtain a more generalized view of the effects of these type of contaminants towards ecosystems.
